# Impact and sustainability of centralising acute stroke services in English metropolitan areas: retrospective analysis of hospital episode statistics and stroke national audit data

**DOI:** 10.1136/bmj.l1

**Published:** 2019-01-23

**Authors:** Stephen Morris, Angus I G Ramsay, Ruth J Boaden, Rachael M Hunter, Christopher McKevitt, Lizz Paley, Catherine Perry, Anthony G Rudd, Simon J Turner, Pippa J Tyrrell, Charles D A Wolfe, Naomi J Fulop

**Affiliations:** 1 Department of Applied Health Research, University College London, London WC1E 7HB, UK; 2 Alliance Manchester Business School, University of Manchester, Manchester M15 6PB, UK; 3 Research Department of Primary Care and Population Health, University College London, London NW3 2PF, UK; 4 Department of Population Health Sciences, School of Population Heath and Environmental Sciences, King’s College London, London SE1 1UL, UK; 5 Stroke Programme, Royal College of Physicians, London, UK; 6 Guy’s and St Thomas’ NHS Foundation Trust, St Thomas' Hospital, London SE1 7EH, UK; 7 Health Policy, Politics and Organisation (HiPPO) Research Group, Centre for Primary Care, School of Health Sciences, University of Manchester, Manchester M13 9PL, UK; 8 Stroke and Vascular Centre, University of Manchester, Manchester Academic Health Science Centre, Salford Royal Hospitals NHS Foundation Trust, Salford M6 8HD, UK; 9 National Institute of Health Research Comprehensive Biomedical Research Centre, Guy’s and St Thomas’ NHS Foundation Trust and King’s College London, Guy’s Hospital, London SE1 9RT, UK

## Abstract

**Objectives:**

To investigate whether further centralisation of acute stroke services in Greater Manchester in 2015 was associated with changes in outcomes and whether the effects of centralisation of acute stroke services in London in 2010 were sustained.

**Design:**

Retrospective analyses of patient level data from the Hospital Episode Statistics (HES) database linked to mortality data from the Office for National Statistics, and the Sentinel Stroke National Audit Programme (SSNAP).

**Setting:**

Acute stroke services in Greater Manchester and London, England.

**Participants:**

509 182 stroke patients in HES living in urban areas admitted between January 2008 and March 2016; 218 120 stroke patients in SSNAP between April 2013 and March 2016.

**Interventions:**

Hub and spoke models for acute stroke care.

**Main outcome measures:**

Mortality at 90 days after hospital admission; length of acute hospital stay; treatment in a hyperacute stroke unit; 19 evidence based clinical interventions.

**Results:**

In Greater Manchester, borderline evidence suggested that risk adjusted mortality at 90 days declined overall; a significant decline in mortality was seen among patients treated at a hyperacute stroke unit (difference-in-differences −1.8% (95% confidence interval −3.4 to −0.2)), indicating 69 fewer deaths per year. A significant decline was seen in risk adjusted length of acute hospital stay overall (−1.5 (−2.5 to −0.4) days; P<0.01), indicating 6750 fewer bed days a year. The number of patients treated in a hyperacute stroke unit increased from 39% in 2010-12 to 86% in 2015/16. In London, the 90 day mortality rate was sustained (P>0.05), length of hospital stay declined (P<0.01), and more than 90% of patients were treated in a hyperacute stroke unit. Achievement of evidence based clinical interventions generally remained constant or improved in both areas.

**Conclusions:**

Centralised models of acute stroke care, in which all stroke patients receive hyperacute care, can reduce mortality and length of acute hospital stay and improve provision of evidence based clinical interventions. Effects can be sustained over time.

## Introduction

Stroke is a leading cause of mortality and disability.^1^ A wealth of evidence shows that organised care in an inpatient stroke unit is associated with better quality of care and reduced death and dependency.^2^ ^3^ ^4^ Evidence also suggests that variation exists within and between countries in both access to stroke unit care and organisational models within stroke units,^5^ ^6^ which affects access to rapid diagnosis and treatment, secondary prevention, and rehabilitation.^7^ In several countries, acute stroke services are being centralised into “hub and spoke” systems as a means of improving access to organised care in inpatient stroke units. Hospitals providing different levels of stroke care work together to create a centralised system,^8^ in which all patients in the system with acute stroke are taken to central specialist units rather than the nearest hospital, with a larger number of hospitals providing care beyond the acute phase. Research in several countries including the US, Canada, the Netherlands, Denmark, and Australia suggests that this approach may improve provision of evidence based care processes for all stroke patients, by increasing access to specialist care and thrombolysis where appropriate.^9^ ^10^ ^11^ ^12^ ^13^ ^14^ Although improved clinical outcomes among patients admitted to organised inpatient stroke care are well documented, relatively little evidence exists to show whether centralising acute stroke care to a small number of high volume specialist centres produces better clinical outcomes at the system level across all stroke patients.^15^ ^16^ Our previous research showed reductions in mortality after the centralisation of stroke services in London (see below). A recent study found that centralisation in the Central Denmark Region significantly reduced length of acute hospital stay without compromising quality, and mortality and readmissions remained unchanged.^17^ No studies have evaluated longer term sustainability of the effects of centralising acute stroke services.

In 2010 acute stroke services were centralised across two metropolitan areas of England—Greater Manchester (population 2.7 million) and London (8.2 million).^18^ Our earlier analysis using data from January 2008 to March 2012 showed that in London, where hyperacute stroke care was provided to all patients, a reduction in mortality and length of acute hospital stay occurred; in Greater Manchester, where specialist hyperacute stroke care was provided only to patients presenting within four hours of developing stroke symptoms, no effect on mortality was seen but length of hospital stay decreased.^19^ We also showed that patients treated at hyperacute stroke units (HASUs) in Greater Manchester and London were significantly more likely to receive evidence based interventions than those treated at other units.^20^ In Greater Manchester, 39% of stroke patients were admitted to a HASU, compared with 93% in London, possibly explaining the difference in mortality outcomes between the two areas. Both centralisations had a high probability of being cost effective.^21^ Other research has shown that outcomes for patients treated at HASUs in London improved after the centralisation, whereas outcomes worsened for those in London not treated at HASUs^22^; differences in in-hospital mortality between weekday and weekend admissions fell in London between 2008 and 2014, but this was not related to the centralisation^23^; and the centralisation in London was cost effective.^24^

In Greater Manchester, further centralisation of the acute stroke system was implemented in April 2015; also, few data are available on the sustainability of the effects of centralising acute stroke services. Given this, plus the growing interest in reconfiguration of services generally,^25^ we extended our original analyses with new data covering the period to March 2016. The aims of this study were to investigate whether the further centralisation of acute stroke services in Greater Manchester in 2015 had an effect on outcomes and whether the effects of the 2010 centralisation in London were sustained over an additional four years (2012-16). We also analysed provision of evidence based interventions, as these may explain outcomes.^20^

## Methods

### The centralisations

**Fig 1:**
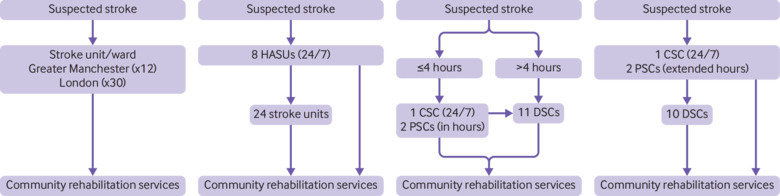
Simplified pre-centralisation and post-centralisation models in London and Greater Manchester. First panel: before 2010 centralisations in London and Greater Manchester. Second panel: after 2010 centralisation in London. Third panel: after 2010 centralisation in Greater Manchester until 2015. Fourth panel: after 2015 centralisation in Greater Manchester. CSC=comprehensive stroke centre; DSC=district stroke centre; HASU=hyperacute stroke unit; PSC=primary stroke centre.

Before the centralisations, in both London and Greater Manchester, patients with suspected stroke were taken to the nearest emergency department to receive stroke care and then treated on a stroke unit or general ward (fig 1, first panel). After the centralisation in London in 2010, 24/7 specialist care was provided to all patients in eight designated HASUs. All patients with suspected stroke (having a deficit on one or more of the face, arm, and speech elements of the FAST test) were taken by the London Ambulance Service to the HASU that involved the shortest journey time (no more than 30 minutes away). The appropriate HASU for a particular postcode may change depending on traffic conditions. Ambulance staff may not necessarily transport patients to the nearest HASU depending on capacity issues. On arrival at the HASU, patients are assessed immediately by specialised stroke medical teams capable of rapid brain imaging and thrombolysis when appropriate. Patients are then admitted to a HASU bed where they receive hyperacute care for up to 72 hours after admission. Once patients are stabilised, they are either transferred to one of 24 stroke units that provide acute rehabilitation services and ongoing medical supervision or discharged home and receive community rehabilitation services (fig 1, second panel).^18^ The location of the stroke unit that patients are taken to depends on their postcode of residence; patients are usually transferred to a stroke unit close to their home, which may be in the same hospital as the HASU. Length of stay in the stroke unit varies, lasting until the patient is well enough to be discharged from an acute inpatient setting. Following their stay in the stroke unit, patients are discharged home or to a specialist inpatient facility for long term rehabilitation. After the 2010 reconfiguration in Greater Manchester (until 2015), patients presenting within four hours of developing stroke symptoms were taken directly to a HASU (one 24/7 HASU called a comprehensive stroke centre (CSC) and two HASUs running 7am-7pm, Monday-Friday (primary stroke centres; PSCs)); all other patients were taken to one of 10 district stroke centres (DSCs), designated to provide all aspects of post-thrombolysis stroke care (fig 1, third panel).^26^ After the 2015 centralisation in Greater Manchester, all patients were eligible for treatment in a HASU rather than just those arriving at hospital within four hours, and the two HASUs operating “in hours” extended the hours in which they would admit stroke patients (7am-11pm, Monday-Sunday) (fig 1, fourth panel). The HASUs in Greater Manchester operate similarly to those in London.

### Data

#### Analysis of mortality and length of acute hospital stay

To investigate mortality and length of acute hospital stay, we obtained patient level data from the Hospital Episode Statistics (HES) database for all patients in England with a primary diagnosis of stroke defined using international classification of diseases, 10th revision (ICD-10) codes I61 (intracerebral haemorrhage), I63 (cerebral infarction), or I64 (stroke, not specified as haemorrhage or infarction, which usually refers to cases in which the cause of the stroke has not been documented in the medical record) between 1 January 2008 and 31 March 2016 (that is, 33 quarters/99 months).^27^ We excluded subarachnoid haemorrhage (ICD-10 code I60) as it is managed through a different clinical pathway.^28^ The dataset was confined to patients living in urban areas (defined as “urban-less sparse” using the urban/rural classification for England).^29^ We linked the data to mortality data supplied by the Office for National Statistics,^30^ using an anonymised unique patient identifier to identify deaths from any cause and at any place of death (hospital or otherwise) by 90 days after hospital admission. We measured length of acute hospital stay (HASU plus stroke unit stay) in days as the difference between date of admission and date of discharge, including same day transfers between hospitals. We created regional variables to identify whether the hospital trust the patient was admitted to was in London, Greater Manchester, or the rest of England. The start of our analysis period was January 2008, following the publication of the National Stroke Strategy for the English NHS in December 2007.^31^ The centralisations in Greater Manchester were completed in April 2010 and March/April 2015. The centralisation in London began to be implemented in October 2009 and was fully operational in July 2010. Data were available for 509 182 admissions, of which 34 051 were in Greater Manchester (9412 from January 2008 to March 2010, 20 390 from April 2010 to March 2015, 4249 from April 2015 to March 2016), 69 847 were in London (18 654 before the centralisation, 51 193 after), and 405 284 were for the rest of England. Mortality data for our analysis were complete; we dropped 17 411 (3%) observations from the analysis of length of acute hospital stay because admission or discharge data were missing (4207 observations) or the acute hospital stay lasted more than 120 days (13 204). 

#### Analysis of evidence based clinical interventions

To investigate evidence based clinical interventions, we used patient level data collected for the clinical audit component of the Sentinel Stroke National Audit Programme (SSNAP),^32^ organised by the Royal College of Physicians. Our data covered the period 1 April 2013 to 31 March 2016 and included data submitted by every stroke unit in England. We examined whether the patient was treated in a HASU, plus 19 binary clinical interventions (measured from time of hospital arrival or onset of stroke symptoms for patients who were already in hospital): brain scan within 60 minutes, 180 minutes, or 24 hours; administration of intravenous thrombolysis (tissue plasminogen activator) if eligible, and whether it was given within 60 minutes; swallow screen within four hours; admission to a stroke unit within four hours; assessment by a stroke consultant physician within 14 hours or 24 hours; assessment by a stroke nurse within 12 hours or 24 hours; assessment by a specialist physiotherapist within 24 hours or 72 hours; assessment by an occupational therapist within 24 hours or 72 hours; formal swallow assessment by a speech and language therapist within 24 hours or 72 hours; and communication assessment by a speech and language therapist within 24 hours or 72 hours. These measures are quality interventions routinely reported by SSNAP. Data were available for 218 120 admissions, of which 12 086 were in Greater Manchester (7606 from April 2013 to March 2015, 4480 from April 2015 to March 2016), 24 281 were in London, and 181 753 were for the rest of England.

### Statistical analyses

We evaluated whether the 2015 centralisation of acute stroke services in Greater Manchester had an effect on mortality and length of hospital stay by using a between region difference-in-differences regression analysis comparing the change over time in Greater Manchester with the change over time in the rest of England (controlling for outcomes in London). We did the analysis at hospital level, using monthly observations of risk adjusted mortality and length of hospital stay. The risk adjustment was done at the patient level. We calculated expected risks of death at 90 days after admission by using patient level logistic regressions, including binary indicators for sex and age interactions (age measured in five year bands), stroke diagnosis using the first four digits of the primary ICD-10 diagnostic code (19 categories), number of comorbidities derived from secondary ICD-10 diagnostic codes, presence of 16 comorbidities based on ICD-10 chapter headings derived from secondary ICD-10 diagnostic codes, ethnic group (19 categories), and fifth of deprivation of the lower layer super output areas in which the patient lived.^33^ We ran the patient level regressions only for patients who had a stroke before the centralisations so that risk adjustment was not contaminated by the changes. We used the regression coefficients (derived from the logistic regressions for the pre-implementation period) to predict the probability of mortality for every patient (in both pre-implementation and post-implementation periods). We aggregated these to create a dataset of the actual percentage of patients who died and the expected percentage by admitting hospital and month. We tested whether the 2015 centralisation had an effect on mortality by using least squares regression of the actual minus expected mortality percentage against interaction terms between Greater Manchester and the post-April 2015 centralisation period. We included binary indicators for admitting hospital (520 hospital fixed effects) and month (99 time fixed effects), as well as an interaction term between London and the post-July 2010 London centralisation period to control for the effects of the London reconfiguration. We used a similar method in our previous study,^19^ and before that it was used in an evaluation of the advancing quality initiative in the north west of England.^34^ Some hospitals began to reconfigure their services before April 2015, and we controlled for this in our analysis by using hospital and time fixed effects. We did not include a separate interaction term for the April 2010 centralisation in Greater Manchester because our previous analysis showed no effect of this reconfiguration on mortality. Each observation was weighted by the number of patients treated at that hospital in that month. We re-ran the second stage of the difference-in-differences analysis including an interaction term only for the HASUs and the post-centralisation period (note that the primary stroke centres were not 24/7 HASUs, but data on time of arrival at the hospital were not available so the analysis included all patients treated at these hospitals irrespective of when they arrived). We ran all of the above separately for stroke diagnoses (ICD-10 codes I61, I63, and I64).

We used the same approach for length of hospital stay in Greater Manchester, but our risk adjustment equation was estimated using a generalised linear model with γ family and log link to account for data skewness.^35^ We also added binary indicators for mortality at three, 30, and 90 days after stroke to the risk equation and used the regression coefficients to predict expected mean length of acute hospital stay. We included a separate interaction term for the pre-April 2010 centralisation in Greater Manchester because our previous analysis showed a significant effect of the 2010 centralisation on length of hospital stay; our comparison was therefore of the change in length of stay following the second centralisation compared with the first.

To analyse whether the effects of the 2010 centralisation in London were sustained over the period 2012-16, using patient level data for London only we regressed 90 day mortality against the covariates used in the risk adjustment model described above, plus hospital and month. We used logistic regression analysis. We then calculated the predictive margins of month on mortality and plotted these on a graph. We tested for significant variations in adjusted mortality since the centralisation by using Wald tests, and we present the results as P values under the null hypothesis that the regression coefficient for every month after the centralisation (which was fully operational from July 2010) was the same as the regression coefficient for July 2010. In the analysis of length of hospital stay in London, we used a similar approach except that we used the generalised linear model as above and also controlled for mortality at three, 30, and 90 days.

For the analysis of the evidence based clinical interventions, we used all data submitted across England and regressed whether or not each of the clinical interventions had been achieved against sex, age (measured in five year bands), stroke diagnosis (cerebral infarction or intracerebral haemorrhage), whether the stroke happened inside or outside hospital, and worst level of consciousness on arrival at the hospital (alert, verbally arousable, not alert, or totally unresponsive).^36^ We used logistic regression analysis and included interactions terms between time period (1 April 2013 to 31 March 2014, 1 April 2014 to 31 March 2015, 1 April 2015 to 31 March 2016) and region (Greater Manchester, London, rest of England), and we calculated predictive margins for each of these interaction terms to give the adjusted probability that each clinical intervention would be achieved in each region and year, controlling for the covariates. We did not include hospital fixed effects as these were perfectly correlated with region. In Greater Manchester, we were interested in whether achievement of the clinical interventions improved in the third year of our data beyond the changes seen in the rest of England. In London, we were interested in whether the achievement of the clinical interventions was constant over the three year period. For comparison, we present the findings alongside the results of our original analyses covering the period 2010-12, which included fewer clinical interventions.^20^

### Patient and public involvement

This study was supported by a patient and families research advisory group that was consulted on the original research question and methods for disseminating the outputs of this study. We also presented findings from previous analyses done as part of this study to these groups to ensure that we were addressing questions and communicating lessons in a meaningful way. The findings of this research will be disseminated to the relevant patient community in an accessible way.

## Results

### Outcomes

Across all stroke types combined, unadjusted mortality at 90 days in Greater Manchester fell by 3.1 percentage points after the April 2015 centralisation; in the rest of England, the reduction was 2.0% (table 1). The unadjusted between region difference-in-differences indicate an additional 1.1% reduction in 90 day mortality in Greater Manchester over and above the reduction seen in the rest of England.

**Table 1:** Unadjusted between region difference-in-differences in mortality at 90 days (all stroke types; Greater Manchester versus rest of England, excluding London)

Analysis	No of admissions	Mortality (%)
Rest of England (January 2008 to March 2015)	356 841	23.3
Rest of England (April 2015 to March 2016)	48 443	21.3
Greater Manchester (January 2008 to March 2015)	29 802	22.7
Greater Manchester (April 2015 to March 2016)	4249	19.6
Differences:		
Rest of England (April 2015 to March 2016) minus (January 2008 to March 2015)	-	−2.0
Greater Manchester (April 2015 to March 2016) minus (January 2008 to March 2015)	-	−3.1
Difference-in-differences:		
Greater Manchester minus rest of England	-	−1.1

Values are based on proportion of patients who died in each region in each time period.

We found borderline evidence of adjusted between region difference-in-differences in 90 day mortality in Greater Manchester across all types of stroke combined (95% confidence interval −2.7 to 0.01; table 2). When we re-ran the analysis only for patients treated at a HASU (86% of all stroke patients, see below), we saw a 1.8% (−3.4 to −0.2) reduction in 90 day mortality (table 2); the coefficient was larger (more negative) than in the all hospital model and significantly different from zero. Assuming an estimated 4500 strokes in Greater Manchester each year (see above), and that 86% of these were treated in a HASU (see below), the mortality reduction is consistent with 69 fewer deaths per year (4500×0.86×−1.8/100).

**Table 2:** Adjusted between region difference-in-differences (Greater Manchester versus rest of England and HASUs in Greater Manchester versus rest of England, controlling for London). Values are absolute differences in risk adjusted mortality at 90 days after admission

Type of stroke	Rest of England			Greater Manchester			HASUs in Greater Manchester			Difference-in-differences (95% CI)	
	Before	After		Before	After		Before	After		Greater Manchester *v* rest of England	HASUs in Greater Manchester *v* rest of England
All stroke types	23.4	20.8		22.8	18.6		23.5	18.4		−1.3 (−2.7 to 0.01)	−1.8 (−3.4 to −0.2)
Intracerebral haemorrhage (I61*)	41.9	43.4		42.6	39.7		43.1	39.9		−4.1 (−8.4 to 0.2)	−5.0 (−10.3 to 0.4)
Cerebral infarction (I63*)	19.9	17.0		19.8	15.8		20.5	15.4		−1.2 (−2.6 to 0.2)	−1.9 (−3.6 to −0.3)
Stroke, not specified as haemorrhage or infarction (I64*)	24.4	19.3		21.6	16.3		22.7	19.7		−1.0 (−5.8 to 3.7)	4.6 (−3.8 to 12.9)

HASU=hyperacute stroke unit.

Values in difference-in-differences columns are absolute differences in risk adjusted mortality showing change over time in Greater Manchester minus change over time in rest of England (see text). Values in preceding columns show absolute risk adjusted mortality in rest of England, Greater Manchester, and Greater Manchester HASUs. To estimate these, separate models were run for rest of England, Greater Manchester, and Greater Manchester HASUs. Patient level data were used to regress mortality against sex and age interactions, stroke diagnosis using first four digits of primary ICD-10 diagnostic code, number of comorbidities derived from secondary ICD-10 diagnostic codes, presence of 16 comorbidities based on ICD-10 chapter headings derived from secondary ICD-10 diagnostic codes, ethnic group, fifth of deprivation, hospital and calendar month, plus indicator variables for whether patient was admitted before or after April 2015 reconfiguration in Greater Manchester; predictive margins for this indicator variable are shown in table. Values in difference-in-differences columns do not equal differences in absolute risk adjusted mortality in preceding columns: with statistical method used to estimate difference-in-differences (see text), it is not possible to recover absolute risk adjusted mortality levels directly.

* ICD-10 codes.

In Greater Manchester, mean unadjusted length of acute hospital stay across all stroke types was 17.4 days between April 2010 and March 2015 and 14.4 days after the 2015 centralisation; figures in the rest of England were 18.1 days and 15.7 days, respectively (table 3). The unadjusted between region difference-in-differences in length of hospital stay was −0.6 days.

**Table 3:** Unadjusted between region difference-in-differences in length of acute hospital stay (all stroke types; Greater Manchester versus rest of England, excluding London)

Analysis	No of admissions	Unadjusted length of hospital stay (mean days)
Rest of England (January 2008 to March 2010)	105 456	21.0
Rest of England (April 2010 to March 2015)	240 369	18.1
Rest of England (April 2015 to March 2016)	47 507	15.7
Greater Manchester (January 2008 to March 2010)	8862	21.7
Greater Manchester (April 2010 to March 2015)	18 994	17.4
Greater Manchester (April 2015 to March 2016)	4054	14.4
Differences:		
Rest of England (April 2015 to March 2016) minus (April 2010 to March 2015)	-	−2.4
Greater Manchester (April 2015 to March 2016) minus (April 2010 to March 2015)	-	−3.0
Difference-in-differences:		
Greater Manchester minus rest of England	-	−0.6

Values are based on mean length of stay in days in each region in each time period.

Across all stroke types, we found a significantly larger decline in risk adjusted length of acute hospital stay in Greater Manchester compared with the rest of England, by 1.5 (−2.5 to −0.4) days (table 4). If this reduction was applied to the estimated 4500 patients with a stroke in Greater Manchester each year, this would result in 6750 fewer bed days a year.

**Table 4:** Adjusted between region difference-in-differences in risk adjusted length of acute hospital stay (Greater Manchester versus rest of England, controlling for London)

Type of stroke	Rest of England		Greater Manchester		Difference-in-differences (95% CI): Greater Manchester *v* rest of England
	Before	After	Before	After	
All stroke subtypes	19.7	15.7	19.6	13.2	−1.5 (−2.5 to −0.4)
Intracerebral haemorrhage (I61*)	19.8	16.9	19.7	15.9	−1.2 (−3.3 to 1.0)
Cerebral infarction (I63*)	20.4	16.2	21.0	13.5	−1.3 (−2.4 to −0.2)
Stroke, not specified as haemorrhage or infarction (I64*)	15.9	12.0	15.0	11.6	−4.4 (−7.0 to −1.8)

Values in difference-in-differences columns are absolute differences in risk adjusted length of acute hospital stay showing change over time in Greater Manchester minus change over time in rest of England (see text). Values in preceding columns show absolute risk adjusted length of hospital stay in rest of England and Greater Manchester. To estimate these, separate models were run for rest of England and Greater Manchester. Patient level data were used to regress length of hospital stay against sex and age interactions, stroke diagnosis using first four digits of primary ICD-10 diagnostic code, number of comorbidities derived from secondary ICD-10 diagnostic codes, presence of 16 comorbidities based on ICD-10 chapter headings derived from secondary ICD-10 diagnostic codes, ethnic group, fifth of deprivation, hospital and calendar month, mortality at 3, 30, and 90 days, plus indicator variables for whether patient was admitted before or after April 2015 reconfiguration in Greater Manchester; predictive margins for this indicator variable are shown in table. Values in difference-in-differences columns do not equal differences in absolute risk adjusted length of hospital stay in preceding columns: with statistical method used to estimate difference-in-differences (see text), it is not possible to recover absolute risk adjusted length of hospital stay levels directly.

* ICD-10 codes.

We re-ran our models on patients stratified by type of stroke (see supplementary table A for further details of the numbers of patients with each type of stroke in each time period and region), and found that reductions in mortality and length of hospital stay were largely achieved among patients diagnosed as having cerebral infarction (table 2 and table 4), who were the majority of cases (supplementary table A). For length of acute hospital stay, the largest decline was in the I64 (stroke unspecified) subgroup, which may be a function of better access to diagnostic testing on arrival at the hospital (especially in patients admitted to a HASU), and, as a consequence of this, declining numbers of patients falling into this group (supplementary table A). Mortality and length of stay outcomes for patients with intracerebral haemorrhage were non-significant, possibly owing to the relatively small numbers of patients in the subgroup (supplementary table A).

**Fig 2:**
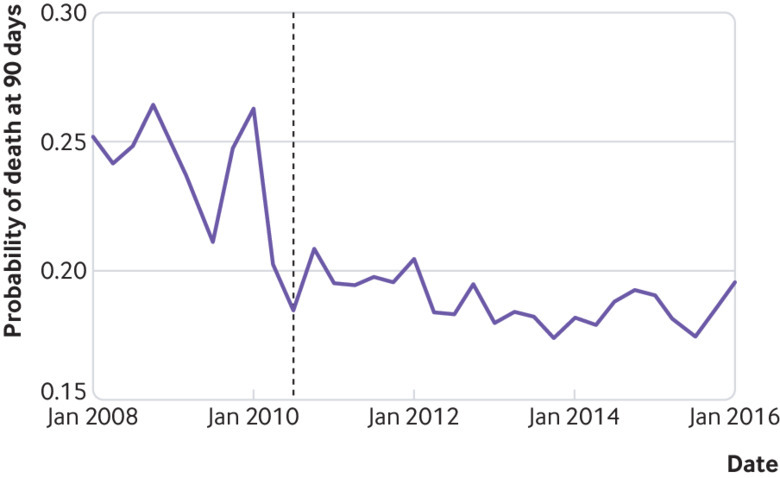
Adjusted trends in mortality at 90 days in London. Note that y axis does not start at zero. Vertical line indicates when centralisation in London was fully operational (July 2010), although centralisation began to be implemented in October 2009. P value (under null hypothesis that regression coefficient for every month after centralisation (which occurred in July 2010) is same as regression coefficient for July 2010) is 0.09

**Fig 3:**
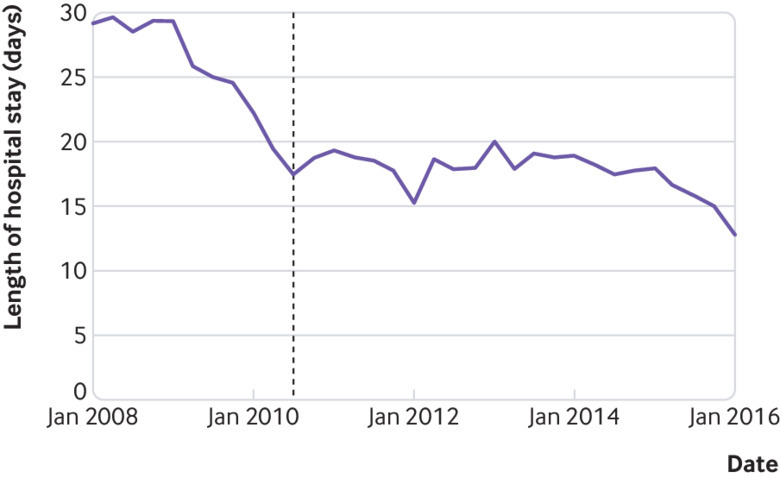
Adjusted trends in length of hospital stay in London. Vertical line indicates when centralisation in London was fully operational (July 2010), although centralisation began to be implemented in October 2009. P value (under null hypothesis that regression coefficient for every month after centralisation (which occurred in July 2010) is same as regression coefficient for July 2010) is <0.01)

In London, we found no significant variation in mortality at 90 days over time since the centralisation (P=0.09; fig 2). Length of acute hospital stay since the centralisation significantly declined (P<0.01; fig 3).

### Treatment in hyperacute stroke unit

In Greater Manchester, the percentage of patients treated in a HASU increased from 39% in 2010-12 to 57% in 2013/14, to 64% in 2014/15, and to 86% in 2015/16 (supplementary table B). Equivalent figures for patients in London treated in a HASU were 93%, 95%, 94%, and 94%, respectively.

### Evidence based clinical interventions

In Greater Manchester, achievement of evidence based clinical interventions after the 2015 centralisation either stayed the same or improved compared with the previous two years (table 5). Improvements of greater than 10% occurred for several “front door” processes (brain scan within 60 minutes, admitted to a stroke unit within four hours) and assessments over the first 72 hours (assessment by a stroke consultant physician within 14 and 24 hours, assessment by a specialist physiotherapist with 24 hours, assessment by an occupational therapist within 24 hours, and communication assessment by a speech and language therapist within 24 hours). Improvements and levels of achievement in the rest of England during the same time period were either similar to or lower than those seen in Greater Manchester. In London the “front door” processes, use of thrombolysis, and assessments during the first 72 hours all stayed high and constant over the three years with no more than a 5% fluctuation each year (table 6). The percentage of patients admitted to a stroke unit within four hours and the percentage having a formal swallow assessment by a speech and language therapist within 72 hours fell by 5-10%. We identified similar trends when we used unadjusted data (supplementary tables C-E).

**Table 5:** Adjusted percentages of patients receiving clinical interventions in Greater Manchester and rest of England by year. Values are predictive margins

Intervention	Greater Manchester: risk to adjusted proportions (95% CI)					Rest of England: risk to adjusted proportions (95% CI)		
	Ramsay*	2013/14	2014/15	2015/16		2013/14	2014/15	2015/16
Brain scan within 60 min		40.7 (39.1 to 42.2)	49.2 (47.6 to 50.8)	60.8 (59.4 to 62.2)		43.0 (42.6 to 43.4)	44.8 (44.4 to 45.2)	47.6 (47.3 to 48.0)
Brain scan within 180 min	65.2 (64.3 to 66.2)	74.1 (72.7 to 75.4)	81.7 (80.5 to 82.9)	86.4 (85.4 to 87.3)		70.9 (70.6 to 71.3)	74.0 (73.7 to 74.3)	77.0 (76.7 to 77.3)
Brain scan within 24 h	94.0 (93.5 to 94.4)	95.1 (94.4 to 95.8)	97.1 (96.5 to 97.6)	97.4 (96.9 to 97.9)		94.9 (94.8 to 95.1)	96.0 (95.8 to 96.1)	96.7 (96.6 to 96.8)
tPA to eligible patients		67.9 (63.6 to 72.1)	82.3 (78.1 to 86.5)	88.6 (85.5 to 91.7)		80.6 (79.8 to 81.3)	89.1 (88.5 to 89.7)	91.8 (91.3 to 92.4)
tPA within 60 min		74.4 (69.6 to 79.2)	70.5 (65.1 to 75.8)	74.6 (70.2 to 78.9)		53.1 (52.0 to 54.2)	57.1 (56.0 to 58.1)	59.7 (58.6 to 60.7)
Swallow screen within 4 h		58.7 (57.1 to 60.3)	63.4 (61.8 to 65.0)	69.2 (67.8 to 70.6)		59.7 (59.3 to 60.1)	63.9 (63.5 to 64.2)	67.7 (67.3 to 68.0)
Admitted to SU within 4 h	55.9 (54.9 to 57.0)	57.4 (55.8 to 59.1)	58.8 (57.1 to 60.5)	79.1 (77.9 to 80.4)		51.3 (50.9 to 51.7)	52.8 (52.4 to 53.2)	53.4 (53.0 to 53.7)
Consultant assessment 14 h		57.6 (55.9 to 59.2)	58.9 (57.2 to 60.6)	79.2 (78.0 to 80.4)		50.6 (50.2 to 51.0)	52.1 (51.7 to 52.5)	52.6 (52.2 to 53.0)
Consultant assessment 24 h		78.2 (76.9 to 79.6)	80.2 (78.9 to 81.5)	93.0 (92.3 to 93.8)		81.1 (80.8 to 81.4)	82.8 (82.5 to 83.1)	84.3 (84.1 to 84.6)
Stroke nurse assessment 12 h		86.4 (85.3 to 87.4)	86.6 (85.5 to 87.7)	91.2 (90.4 to 92.0)		86.5 (86.2 to 86.7)	88.1 (87.8 to 88.3)	89.3 (89.0 to 89.5)
Stroke nurse assessment 24h		93.4 (92.7 to 94.2)	92.8 (92.0 to 93.6)	94.6 (93.9 to 95.2)		93.2 (93.0 to 93.4)	93.9 (93.7 to 94.0)	94.5 (94.4 to 94.7)
Physiotherapist assessment 24 h		55.4 (53.7 to 57.0)	63.2 (61.6 to 64.8)	80.7 (79.5 to 81.9)		55.6 (55.2 to 56.0)	57.1 (56.7 to 57.4)	59.7 (59.3 to 60.1)
Physiotherapist assessment 72 h	92.1 (91.5 to 92.7)	95.1 (94.3 to 95.8)	97.0 (96.4 to 97.6)	97.5 (97.1 to 98.0)		93.6 (93.4 to 93.8)	93.4 (93.2 to 93.6)	93.7 (93.5 to 93.9)
Occupational therapist 24 h		50.5 (48.8 to 52.2)	61.2 (59.6 to 62.8)	79.4 (78.1 to 80.6)		45.2 (44.7 to 45.6)	47.5 (47.1 to 47.9)	52.3 (51.9 to 52.7)
Occupational therapist 72 h		94.4 (93.6 to 95.1)	96.4 (95.8 to 97.0)	96.9 (96.4 to 97.4)		87.3 (87.1 to 87.6)	88.5 (88.2 to 88.7)	89.7 (89.4 to 89.9)
SaLT swallow 24 h		53.2 (50.8 to 55.6)	56.9 (54.4 to 59.4)	55.7 (53.1 to 58.2)		48.4 (47.8 to 49.0)	49.9 (49.3 to 50.5)	54.0 (53.4 to 54.6)
SaLT swallow 72 h	94.0 (93.6 to 94.4)	85.5 (83.9 to 87.2)	91.3 (89.9 to 92.7)	91.9 (90.6 to 93.3)		78.8 (78.3 to 79.3)	82.2 (81.7 to 82.6)	84.9 (84.5 to 85.3)
SaLT communication 24 h		39.7 (37.3 to 42.2)	53.8 (51.5 to 56.2)	69.9 (68.0 to 71.7)		35.5 (34.9 to 36.1)	38.0 (37.5 to 38.6)	42.0 (41.5 to 42.6)
SaLT communication 72 h		86.7 (85.1 to 88.3)	93.4 (92.2 to 94.5)	96.6 (95.9 to 97.3)		78.3 (77.8 to 78.7)	81.6 (81.2 to 82.0)	85.1 (84.7 to 85.5)

SaLT=speech and language therapist; SU=stroke unit; tPA=tissue plasminogen activator.

* Refers to data from period 2010-12.^20^

See supplementary material for graphical depiction.

**Table 6:** Adjusted percentages of patients receiving clinical interventions in London by year. Values are predictive margins

Intervention	Ramsay*	2013/14	2014/15	2015/16
Brain scan within 60 min		57.3 (56.2 to 58.3)	58.9 (57.9 to 60.0)	59.9 (58.8 to 61.0)
Brain scan within 180 min	66.3 (65.6 to 67.1)	80.0 (79.1 to 80.9)	81.8 (81.0 to 82.6)	82.2 (81.4 to 83.1)
Brain scan within 24 h	95.2 (94.8 to 95.5)	97.2 (96.9 to 97.6)	97.1 (96.8 to 97.5)	97.3 (97.0 to 97.7)
tPA to eligible patients		92.9 (91.6 to 94.2)	94.4 (93.2 to 95.7)	88.6 (86.9 to 90.3)
tPA within 60 min		83.0 (81.0 to 85.0)	83.9 (81.9 to 85.9)	80.8 (78.6 to 83.1)
Swallow screen within 4 h		65.1 (64.1 to 66.2)	68.1 (67.1 to 69.2)	70.6 (69.6 to 71.6)
Admitted to SU within 4 h	66.3 (65.6 to 67.1)	61.1 (60.0 to 62.1)	59.8 (58.7 to 60.8)	60.3 (59.2 to 61.3)
Consultant assessment 14 h		50.3 (49.2 to 51.5)	53.7 (52.6 to 54.8)	51.3 (50.1 to 52.4)
Consultant assessment 24 h		89.3 (88.6 to 90.0)	90.2 (89.6 to 90.9)	88.5 (87.8 to 89.2)
Stroke nurse assessment 12 h		90.8 (90.1 to 91.4)	92.7 (92.1 to 93.2)	93.3 (92.7 to 93.8)
Stroke nurse assessment 24 h		95.5 (95.1 to 96.0)	95.6 (95.2 to 96.1)	96.2 (95.7 to 96.6)
Physiotherapist assessment 24 h		57.4 (56.2 to 58.5)	61.7 (60.5 to 62.8)	64.7 (63.6 to 65.8)
Physiotherapist assessment 72 h	95.4 (95.0 to 95.8)	95.0 (94.5 to 95.5)	96.5 (96.0 to 96.9)	95.8 (95.3 to 96.2)
Occupational therapist 24 h		50.3 (49.1 to 51.4)	57.0 (55.8 to 58.1)	61.5 (60.3 to 62.6)
Occupational therapist 72 h		91.1 (90.5 to 91.8)	94.6 (94.1 to 95.1)	94.1 (93.6 to 94.7)
SaLT swallow 24 h		53.9 (52.2 to 55.7)	54.1 (52.5 to 55.8)	51.5 (49.8 to 53.3)
SaLT swallow 72 h	98.2 (97.9 to 98.4)	89.5 (88.5 to 90.6)	91.8 (90.9 to 92.7)	88.4 (87.3 to 89.5)
SaLT communication 24 h		46.4 (45.0 to 47.8)	50.1 (48.7 to 51.5)	50.7 (49.2 to 52.1)
SaLT communication 72 h		89.7 (88.8 to 90.5)	93.0 (92.3 to 93.7)	91.0 (90.2 to 91.8)

SaLT=Speech and Language Therapist; SU=stroke unit; tPA=tissue plasminogen activator.

* Refers to data from period 2010-12.^20^

See supplementary figures A-C for graphical depiction.

## Discussion

After the further centralisation of stroke care in Greater Manchester in 2015, a larger proportion of patients were treated in a HASU (comprehensive stroke centre or primary stroke centre) than in 2010. Achievement of evidence based clinical interventions either stayed the same or improved compared with the rest of England. A decline in mortality from stroke occurred after the centralisation, and we found borderline evidence that a reduction in mortality occurred across all hospitals in Greater Manchester over and above the changes seen in the rest of England. A reduction in all cause mortality was seen in the HASUs that was statistically significantly larger than the reductions seen elsewhere and would amount to approximately 69 fewer deaths a year. A significant reduction in length of hospital stay of 1.5 days per patient was seen in Greater Manchester over and above the reduction seen in the rest of England, which would amount to approximately 6750 fewer bed days a year. In the analysis for London, mortality and evidence based clinical interventions seem to have varied little over time since the centralisation in July 2010. Length of acute hospital stay since the centralisation significantly declined. This indicates that the improvements seen after the centralisation in London have largely been sustained, with reductions in performance in some clinical interventions and further declines in length of acute hospital stay.

### Strengths and weaknesses of study

The strengths and weakness are similar to those of our earlier studies.^19^ ^20^ The strengths relate to the large national datasets and the robust quasi-experimental analytical frameworks that we used. In addition, our data covered a longer time period allowing us to investigate the effect of further changes in Greater Manchester and whether the improvements in London seen in 2010 to 2012 were maintained over time.

The main limitation is lack of data on severity of stroke; these data are not available in the HES database. If stroke patients were more likely to die before reaching the HASU owing to longer travel distances, or if more patients with less severe strokes were likely to go to hospital after centralisation, this would mean that the level of stroke severity on arrival at the hospital would be different before and after centralisation. As shown in the supplementary material, no discernible differences existed in indicators of stroke severity (worst level of consciousness in first 24 hours after stroke) over time in patients admitted to hospital in Greater Manchester and London. Nevertheless, we cannot rule out the possibility that our findings are due to variations in stroke severity between Greater Manchester, the rest of England, and London over time.

Another limitation is that we were unable to include other important outcomes such as disability. Data on disability and dependence are collected in SSNAP at six month follow-up assessments but are not collected reliably enough for analysis. Additional limitations include lack of data on quality of life, lack of data on pre-hospital processes and outcomes, and limitations in measurement of length of hospital stay when patients may be repatriated between stroke units. Additionally, length of acute hospital stay depends on the availability of community rehabilitation services and the local agreements between acute and rehabilitation services on the timing of transmission of stroke patients from acute care to rehabilitation. Although we controlled for hospital and time specific effects in our analysis, the decrease in length of acute hospital stay may be explained by arrangements for earlier transmission of patients to rehabilitation units from acute stroke services. In addition, the primary stroke centres in Greater Manchester were not 24/7 hyperacute stroke units, but data on time of arrival at the hospital were not available so the analysis included all patients treated at these hospitals irrespective of when they arrived (that is, we included people who were treated at the primary stroke centres even when they were not operating as HASUs), which may mean we have underestimated the impact of centralisation in Greater Manchester. A limitation of our analysis of SSNAP data is that achievement of clinical interventions was measured from time of arrival at hospital rather than from onset of symptoms. Finally, our analysis used data only for patients treated up to one year after the further centralisation in Greater Manchester.

### Strengths and weaknesses in relation to other studies

Our findings extend those of our earlier analyses, examining the longer term effects of the centralisation in London, whether the further centralisation in Greater Manchester was associated with improvements in outcomes, and achievement of clinical interventions. The improvement in outcomes seen in London has been borne out in other studies, although these have also shown that outcomes worsened for patients treated for acute stroke in London but not in a HASU.^22^ Our findings are consistent with a previous analysis based on national stroke audit data showing that patients admitted to stroke services with higher levels of organisation were more likely to receive high quality care and to have a reduced risk of death 30 days after stroke.^2^ Our results are also consistent with those of a study evaluating the centralisation of acute stroke services in Denmark, which used national stroke registry data to show that centralisation reduced length of hospital stay without compromising quality of care.^17^ These studies are important because both were able to control for stroke severity. Our study has focused on one model of acute stroke care—centralised “hub and spoke” systems—but other models are also possible. For example, evidence from a systematic review suggests that for patients with ischaemic stroke the location of initial thrombolysis treatment does not affect outcomes; death and disability rates were not significantly different among thrombolysed patients starting treatment in non-specialist centres versus specialist centres.^37^ The greater travel times in rural areas make centralisation challenging and may necessitate other solutions, such as telemedicine; in Finland, a decentralised telestroke thrombolysis service achieved similar treatment rates and time delays for a rural population to those achieved by a centralised system for an urban population.^38^

### Meaning of study

Our study provides evidence to support the centralisation of acute stroke services in urban areas and shows that this service model should include all stroke patients in those areas. This is supported by our earlier analysis of the changes in London and Greater Manchester, which found that the centralisation in London, where most patients were treated in a HASU, resulted in reductions in mortality, whereas the 2010 Greater Manchester centralisation, in which most patients were not treated in a HASU, did not. This finding is strengthened by our new analysis of the further centralisation in Greater Manchester, in which most patients were treated in a HASU and which showed reductions in mortality. These findings are also supported by data presented here showing the increasing numbers of patients treated at a HASU in Greater Manchester and our analysis of the provision of evidence based clinical interventions (that is, that these were more likely to be achieved after the centralisation in London compared with the first centralisation in Greater Manchester, and that achievement significantly increased with the further centralisation in Greater Manchester and was largely maintained in London).

Our earlier findings on clinical outcomes in London and Greater Manchester were presented in the 2014 NHS Five Year Forward View as evidence of the “case for greater concentration of care,” associated guidance on transforming urgent and emergency care services across the English NHS, and the National Clinical Strategy for Scotland 2016.^19^ ^39^ ^40^ ^41^ Our findings on clinical interventions have been cited in the 2016 edition of the National Clinical Guidance for Stroke as evidence of the benefits of stroke service reorganisation.^20^ ^42^ On the basis of the experiences in London and Greater Manchester, several areas of the UK (including both urban and more rural areas) are considering system-wide reconfiguration of acute stroke services and have cited our earlier research in “case for change” documents; these areas include Yorkshire and the Humber; Birmingham, Solihull, and the Black Country; Sussex; Kent and Medway; South West England; and Cumbria.^43^ ^44^ ^45^ ^46^ ^47^ ^48^ ^49^ Our methods and findings may also inform the centralisation of other healthcare services such as cancer care,^50^ ^51^ cardiac arrest/myocardial infarction,^52^ ^53^ ^54^ major trauma care,^55^ and vascular surgery,^56^ for which evidence of impact is growing.

### Unanswered questions and future research

Given that we could analyse data only for patients treated up to one year after the second centralisation in Greater Manchester, and the fact that SSNAP data indicate that the proportion of patients treated in a HASU and receiving clinical interventions has continued to increase in Greater Manchester,^32^ further research on the longer term effects of the second reconfiguration in Greater Manchester would be beneficial. Future research would also be beneficial to examine effects on disability and dependence after stroke, as well as how and why the improvements seen in London were sustained over time. Given the results of this and our previous study, further research would be helpful to understand how to further improve access to HASU care for all stroke patients. In April 2017 NHS England announced that it will begin commissioning mechanical thrombectomy for patients with certain types of acute ischaemic stroke.^57^ Further work to evaluate the effect of introducing these services into the NHS, and how they ought to be organised, would be useful.

**What is already known on this topic**

In 2010 acute stroke services were centralised across two metropolitan areas of England
In London, where the policy was to provide hyperacute stroke care to all patients, a reduction in mortality and length of hospital stay was seen
In Greater Manchester, where hyperacute stroke care was provided only to patients presenting within four hours of developing symptoms, no impact on mortality was seen but length of hospital stay decreased
Further centralisation of the acute stroke system in Greater Manchester took place in 2015, whereby all stroke patients were eligible for treatment in a hyperacute stroke unit

**What this study adds**

After further centralisation in Greater Manchester, a significant decline in mortality occurred among patients treated at a hyperacute stroke unit and overall length of acute hospital stay fell
Effects of centralisation in London on mortality and length of hospital stay were maintained from 2010 to 2016
This study provides further evidence for centralising acute stroke services in hub and spoke models in urban areas whereby all stroke patients receive hyperacute stroke care

## Web extra

Extra material supplied by authors

**Web appendix:** Supplementary material

## Notes

Contributors: SM, NJF, and AIGR designed the study. SM did the analysis of the Hospital Episodes Statistics data and drafted the manuscript. AIGR did the analysis of the SSNAP data. SM, AIGR, RB, RMH, CM, LP, CP, AGR, SJT, PJT, CDAW, and NJF made substantial contributions to the study design and interpretation of the findings. All authors contributed to the drafting of the article or critical revision for important intellectual content, gave final approval of the version to be published, and agree to be accountable for all aspects of the work in ensuring that questions related to the accuracy or integrity of any part of the article are appropriately investigated and resolved. The corresponding author attests that all listed authors meet authorship criteria and that no others meeting the criteria have been omitted. SM and NJF are the guarantors.

Ethical approval: The study received ethical approval in September 2011 from the London East NHS Research Ethics Committee (ref 11/LO/1396).

Data sharing: No additional data available.

Transparency: The lead author affirms that this manuscript is an honest, accurate, and transparent account of the study being reported; that no important aspects of the study have been omitted; and that any discrepancies from the study as planned (and, if relevant, registered) have been explained.
