# Supplementary material for: Impact and sustainability of centralising acute stroke services in English metropolitan areas: retrospective analysis of hospital episode statistics and stroke national audit data
Source: BMJ. 2019 Jan 23;364:l1. doi: 10.1136/bmj.l1 (PMC6334718; doi:10.1136/bmj.l1)

Supplementary material

Table A. Characteristics of patients before and after centralisation of acute stroke services in Greater Manchester, London and the rest of England

Table B. Patient characteristics in analysis of clinical interventions, by region and year (characteristics from Ramsay et al [2015] included for comparison)

Table C. Unadjusted proportions of patients receiving clinical indicators in Greater Manchester by year

Table D. Unadjusted proportions of patients receiving clinical interventions in London by year

Table E. Unadjusted proportions of patients receiving clinical interventions in Rest of England by year

Figure A. Adjusted likelihood of patients receiving evidence-based clinical interventions in Greater Manchester

Figure B. Adjusted likelihood of patients receiving evidence-based clinical interventions in London

Figure C. Adjusted likelihood of patients receiving evidence-based clinical interventions in London

Table A. Characteristics of patients before and after centralisation of acute stroke services in Greater Manchester, London and the rest of England

	Rest of England			Greater Manchester					London	
	January 2008 to March 2010	April 2010 to March 2015	April 2015 to March 2016	January 2008 to March 2010	April 2010 to March 2015	April 2015 to March 2016	April 2015 to March 2016, not treated in a HASU	April 2015 to March 2016, treated in a HASU	January 2008 to June 2010	July 2010 to March 2016
No. patients	109 795	247 046	48 443	9412	20 390	4249	1400	2849	18 654	51 193
Age, mean (years)	75.7	75.4	75.0	74.3	73.8	73.6	75.2	72.8	73.1	72.8
Female (%)	53.1	51.7	49.7	52.6	50.7	50.0	50.2	49.9	51.0	49.0
White British ethnic group (%)	84.1	85.8	82.8	82.9	84.0	81.9	79.3	83.2	58.5	53.0
Intracerebral hemorrhage (%)†	13.3	13.3	14.2	11.9	12.1	12.3	13.1	11.9	16.4	17.1
Cerebral infarction (%)‡	65.1	75.3	78.9	61.9	68.7	80.1	71.8	85.2	69.4	77.1
Stroke, not specified as hemorrhage or infarction (%)§	22.5	12.3	7.9	26.9	19.9	7.5	15.8	3.4	15.5	7.7
Comorbidities, mean (number)	4.7	5.9	6.5	5.3	6.6	6.9	7.8	6.5	5.1	6.3
Most deprived quintile (%)¶	24.6	23.8	23.2	41.2	39.2	37.2	36.8	37.4	23.2	24.4

† Primary diagnosis of stroke with ICD-10 diagnostic code I61.
‡ Primary diagnosis of stroke with ICD-10 diagnostic code I63.
§ Primary diagnosis of stroke with ICD-10 diagnostic code I64.
¶ Based on 32 482 Lower Layer Super Output Areas of residence in England.

Table B. Patient characteristics in analysis of clinical indicators, by region and year

	Greater Manchester				London				Rest of England			
	**Ramsay**	**2013/14**	**2014/15**	**2015/16**	***Ramsay***	**2013/14**	**2014/15**	**2015/16**	**Ramsay**	**2013/14**	**2014/15**	**2015/16**
**Observations**	10,295	3,877	3,729	4,480	*16,533*	8,131	8,150	8,000	9,044	55,262	62,824	63,667
**Age**												
Mean age (yr)	73.2	73.5	73.7	73.5	*72.7*	72.4	72.5	72.5	73.6	75.3	75.2	75.2
Aged over 75yr (N)		2,077	2,041	2,401		4,276	4,257	4,182		32960	37285	37478
Aged over 75yr (%)	50	53.57	54.73	53.59	*50*	52.59	52.23	52.28	51	59.6	59.4	58.9
**Sex**												
Female (N)		1,974	1,819	2,208		3,969	3,905	3,832		28,009	31,628	31,552
Female (%)	51	50.92	48.78	49.29	*49*	48.81	47.91	47.9	51	50.7	50.3	49.6
**Stroke type**												
Primary infarction (N)		3393	3277	3946		6970	7063	6862		48734	55072	55476
Primary infarction (%)	87	88.38	88.57	88.46	*86*	88.15	87.97	86.73	89	89.4	88.5	87.8
Haemorrhagic (N)		446	423	515		937	966	1050		5772	7174	7732
Haemorrhagic (%)	13	11.62	11.43	11.54	*14*	11.85	12.03	13.27	11	10.6	11.5	12.2
**Where stroke happened**												
Admitted from outside hospital (N)		3,608	3,485	4,195		7,792	7,799	7,671		52,236	59,269	60,168
Admitted from outside hospital (%)	90	93.06	93.46	93.64	*95*	95.83	95.69	95.89	94	94.5	94.3	94.5
**Treatment in HASU**												
Total treated in HASU (N)		2,205	2,389	3,849		7,713	7,668	7,522	-	-	-	-
Total treated in HASU (%)	39	56.9	64.1	85.9	*93*	94.9	94.1	94.0	-	-	-	-

	**Greater Manchester**				**London**				**Rest of England**			
	**Ramsay**	**2013/14**	**2014/15**	**2015/16**	***Ramsay***	**2013/14**	**2014/15**	**2015/16**	**Ramsay**	**2013/14**	**2014/15**	**2015/16**
Reaching hospital in ≤4h (N)		1,254	1,156	1,322		3,060	3,203	3,162	-	21,515	24,381	24,248
Reaching hospital in ≤4h (%)		61.0	59.2	53.8		59.3	59.8	59.1	-	64.0	62.8	60.8
Reaching hospital in ≤48h (N)		1,175	1,077	1,435		2,521	2,190	2,082		17,318	20,050	19,452
Reaching hospital in ≤48h (%)		98.4	98.3	97.8		97.8	97.7	97.8		97.7	97.9	98.1
Treated in HASU if arrive in ≤4h (N)		991	982	1,246		2,999	3,147	3,111	-	-	-	-
Treated in HASU if arrive in ≤4h (%)	67	79.0	85.0	94.3	*96*	98.0	98.3	98.4	-	-	-	-
**Thrombolysis**												
Infarct pts classed as eligible (N)		420	297	382		1432	1344	1284		7889	8103	7573
Infarct pts classed as eligible (%)		12.4	9.1	9.7		20.5	19.0	18.7		16.2	14.7	13.7
Infarct pts receiving tPA (N)		274	244	337		1331	1267	1134		6145	7138	6986
Total receiving tPA (% infarct pts)		8.1	7.4	8.5		19.1	17.9	16.5		12.6	13.0	12.6
Total receiving tPA (% eligible pts)		65.2	82.2	88.2		93.0	94.3	88.3		77.9	88.1	92.3
**Worst level of consciousness**												
Alert (N)		3,230	3,078	3,814		6,829	6,755	6,713		45,988	52,317	53,309
Alert (%)	74	83.31	82.54	85.13	*78*	83.99	82.88	83.91	75	83.2	83.3	83.7
Verbally arousable (N)		331	349	396		795	825	768		5,374	6,108	6,068
Verbally arousable (%)	15	8.54	9.36	8.84	*15*	9.78	10.12	9.6	11	9.7	9.7	9.5
Not alert (N)		181	195	166		317	379	338		2,183	2,585	2,490
Not alert (%)	4	4.67	5.23	3.71	*3*	3.9	4.65	4.23	9	4.0	4.1	3.9
Totally unresponsive (N)		135	107	104		190	191	181		1,717	1,814	1,800
Totally unresponsive (%)	7	3.48	2.87	2.32	*4*	2.34	2.34	2.26	12	3.1	2.9	2.8

‘Ramsay’ refers to the patient characteristics for the post-centralization sample covering the period 2010-2012 reported in Ramsay et al [9]. Patients classified as treated in HASU if treated in HASU at any point in care. Comparator area covered far more of rest of England than in Ramsay et al [9]. HASU= hyperacute stroke unit; yr = year; h = hour; tPA=tissue plasminogen activator; N = number of observations.

Table C. Unadjusted proportions of patients receiving clinical interventions in Greater Manchester by year

Intervention	Ramsay 2015	2013/2014	2014/2015	2015/2016
Scan 60 mins		1490/3839 (38.8%)	1758/3700 (47.5%)	2627/4461 (58.9%)
Scan 180 mins	5785/10295 (56%)	2803/3839 (73.0%)	2998/3700 (81.0%)	3822/4461 (85.7%)
Scan 24h	8814/9563 (92%)	3642/3839 (94.9%)	3587/3700 (97.0%)	4340/4461 (97.3%)
tPA to eligible patients		274/420 (65.2%)	244/297 (82.2%)	337/382 (88.2%)
tPA within 60 mins		197/274 (71.9%)	165/244 (67.6%)	243/337 (72.1%)
Swallow screen in 4h		2050/3544 (57.8%)	2128/3399 (62.6%)	2843/4145 (68.6%)
SU within 4h	4982/10295 (48%)	2414/3870 (62.4%)	2347/3724 (63.0%)	3232/4470 (72.3%)
Consultant assessment 14h		1978/3428 (57.7%)	1977/3346 (59.1%)	3356/4234 (79.3%)
Consultant assessment 24h		2650/3428 (77.3%)	2654/3346 (79.3%)	3925/4234 (92.7%)
Nurse assessment 12h		3136/3664 (85.6%)	3057/3562 (85.8%)	3917/4320 (90.7%)
Nurse assessment 24h		3409/3664 (93.0%)	3289/3562 (92.3%)	4073/4320 (94.3%)
Physio assessment 24h		1927/3498 (55.1%)	2141/3415 (62.7%)	3342/4155 (80.4%)
Physio assessment 72h	8030/8867 (91%)	3315/3498 (94.8%)	3308/3415 (96.9%)	4047/4155 (97.4%)
Occupational therapist 24h		1720/3460 (49.7%)	2059/3419 (60.2%)	3308/4199 (78.8%)
Occupational therapist 72h		3255/3460 (94.1%)	3290/3419 (96.2%)	4063/4199 (96.8%)
SaLT swallow 24h		848/1611 (52.6%)	853/1516 (56.3%)	805/1471 (54.7%)
SaLT swallow 72h	8521/9388 (91%)	1349/1611 (83.7%)	1374/1516 (90.6%)	1342/1471 (91.2%)
SaLT communication 24h		591/1559 (37.9%)	907/1738 (52.2%)	1579/2310 (68.4%)
SaLT communication 72h		1328/1559 (85.2%)	1611/1738 (92.7%)	2222/2310 (96.2%)

‘Ramsay’ refers to the patient characteristics for the post-centralization sample covering the period 2010-2012 reported in Ramsay et al [9].

Table D. Unadjusted proportions of patients receiving clinical interventions in London by year

Intervention	Ramsay	2013/2014	2014/2015	2015/2016
Scan 60 mins		4524/7907 (57.2%)	4733/8029 (59.0%)	4738/7912 (59.9%)
Scan 180 mins	11614/16553 (70%)	6324/7907 (80.0%)	6571/8029 (81.8%)	6508/7912 (82.3%)
Scan 24h	14895/15679 (95%)	7695/7907 (97.3%)	7805/8029 (97.2%)	7709/7912 (97.4%)
tPA to eligible patients		1331/1432 (93.0%)	1267/1344 (94.3%)	1134/1284 (88.3%)
tPA within 60 mins		1104/1331 (83.0%)	1059/1267 (83.6%)	915/1134 (80.7%)
Swallow screen in 4h		5068/7718 (65.7%)	5220/7645 (68.3%)	5369/7606 (70.6%)
SU within 4h	11360/16553 (69%)	4993/8112 (61.6%)	4861/8121 (59.9%)	4806/7973 (60.3%)
Consultant assessment 14h		3932/7821 (50.3%)	4197/7873 (53.3%)	3906/7662 (51.0%)
Consultant assessment 24h		7014/7821 (89.7%)	7118/7873 (90.4%)	6797/7662 (88.7%)
Nurse assessment 12h		7078/7767 (91.1%)	7259/7810 (92.9%)	7178/7678 (93.5%)
Nurse assessment 24h		7443/7767 (95.8%)	7485/7810 (95.8%)	7398/7678 (96.4%)
Physio assessment 24h		4062/7064 (57.5%)	4315/7001 (61.6%)	4498/6970 (64.5%)
Physio assessment 72h	14190/14760 (96%)	6712/7064 (95.0%)	6753/7002 (96.4%)	6670/6970 (95.7%)
Occupational therapist 24h		3386/6766 (50.0%)	3871/6840 (56.6%)	4179/6856 (61.0%)
Occupational therapist 72h		6161/6766 (91.1%)	6464/6840 (94.5%)	6443/6856 (94.0%)
SaLT swallow 24h		1798/3279 (54.8%)	1857/3403 (54.6%)	1600/3104 (51.6%)
SaLT swallow 72h	15431/15684 (98%)	2924/3279 (89.2%)	3114/3403 (91.5%)	2728/3104 (87.9%)
SaLT communication 24h		2269/4933 (46.0%)	2343/4755 (49.3%)	2308/4640 (49.7%)
SaLT communication 72h		4411/4933 (89.4%)	4410/4755 (92.7%)	4204/4641 (90.6%)

‘Ramsay’ refers to the patient characteristics for the post-centralization sample covering the period 2010-2012 reported in Ramsay et al [9]. h=hour; SaLT=Speech and Language Therapist; tPA=tissue plasminogen activator.

Table E. Unadjusted proportions of patients receiving clinical interventions in Rest of England by year

Intervention	2013/2014	2014/2015	2015/2016
Scan 60 mins	22462/54506 (41.2%)	26801/62246 (43.1%)	29160/63208 (46.1%)
Scan 180 mins	38036/54506 (69.8%)	45452/62246 (73.0%)	48277/63208 (76.4%)
Scan 24h	51592/54506 (94.7%)	59642/62246 (95.8%)	61054/63208 (96.6%)
tPA to eligible patients	6145/7889 (77.9%)	7138/8103 (88.1%)	6986/7573 (92.3%)
tPA within 60 mins	2938/6145 (47.8%)	3739/7138 (52.4%)	3901/6986 (55.8%)
Swallow screen in 4h	30570/51864 (58.9%)	37564/59396 (63.2%)	40643/60458 (67.2%)
SU within 4h	33057/55069 (60.0%)	36585/62605 (58.4%)	37599/63441 (59.3%)
Consultant assessment 14h	25968/50422 (51.5%)	30354/57600 (52.7%)	31758/59179 (53.7%)
Consultant assessment 24h	40379/50422 (80.1%)	47114/57600 (81.8%)	49576/59179 (83.8%)
Nurse assessment 12h	44033/51244 (85.9%)	51042/58378 (87.4%)	52874/59613 (88.7%)
Nurse assessment 24h	47616/51244 (92.9%)	54651/58378 (93.6%)	56214/59613 (94.3%)
Physio assessment 24h	26988/48588 (55.5%)	31076/54942 (56.6%)	33301/56297 (59.2%)
Physio assessment 72h	45375/48589 (93.4%)	51115/54943 (93.0%)	52621/56297 (93.5%)
Occupational therapist 24h	20314/45341 (44.8%)	24265/52170 (46.5%)	27830/54192 (51.4%)
Occupational therapist 72h	39408/45341 (86.9%)	45731/52171 (87.7%)	48304/54194 (89.1%)
SaLT swallow 24h	11245/23586 (47.7%)	12416/25219 (49.2%)	13535/24983 (54.2%)
SaLT swallow 72h	18199/23588 (77.2%)	20405/25220 (80.9%)	21103/24983 (84.5%)
SaLT communication 24h	7997/23674 (33.8%)	9620/26621 (36.1%)	11196/27529 (40.7%)
SaLT communication 72h	18049/23674 (76.2%)	21207/26621 (79.7%)	23148/27529 (84.1%)

Ramsay et al [9] did not use Rest of England as a comparator. h=hour; SaLT=Speech and Language Therapist; tPA=tissue plasminogen activator.

Figure A. Adjusted likelihood of patients receiving evidence-based clinical interventions in Greater Manchester

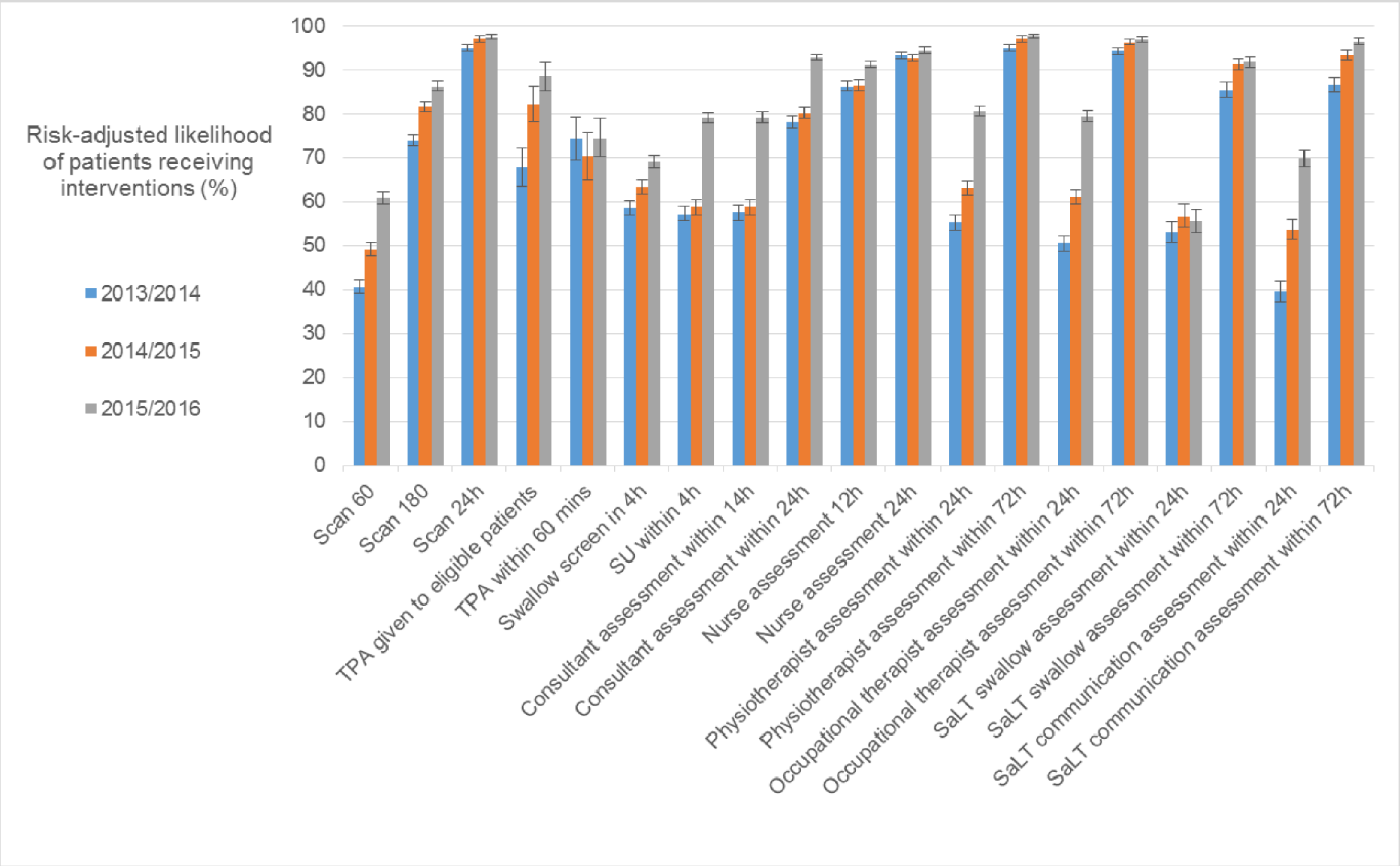

Figure B. Adjusted likelihood of patients receiving evidence-based clinical interventions in London

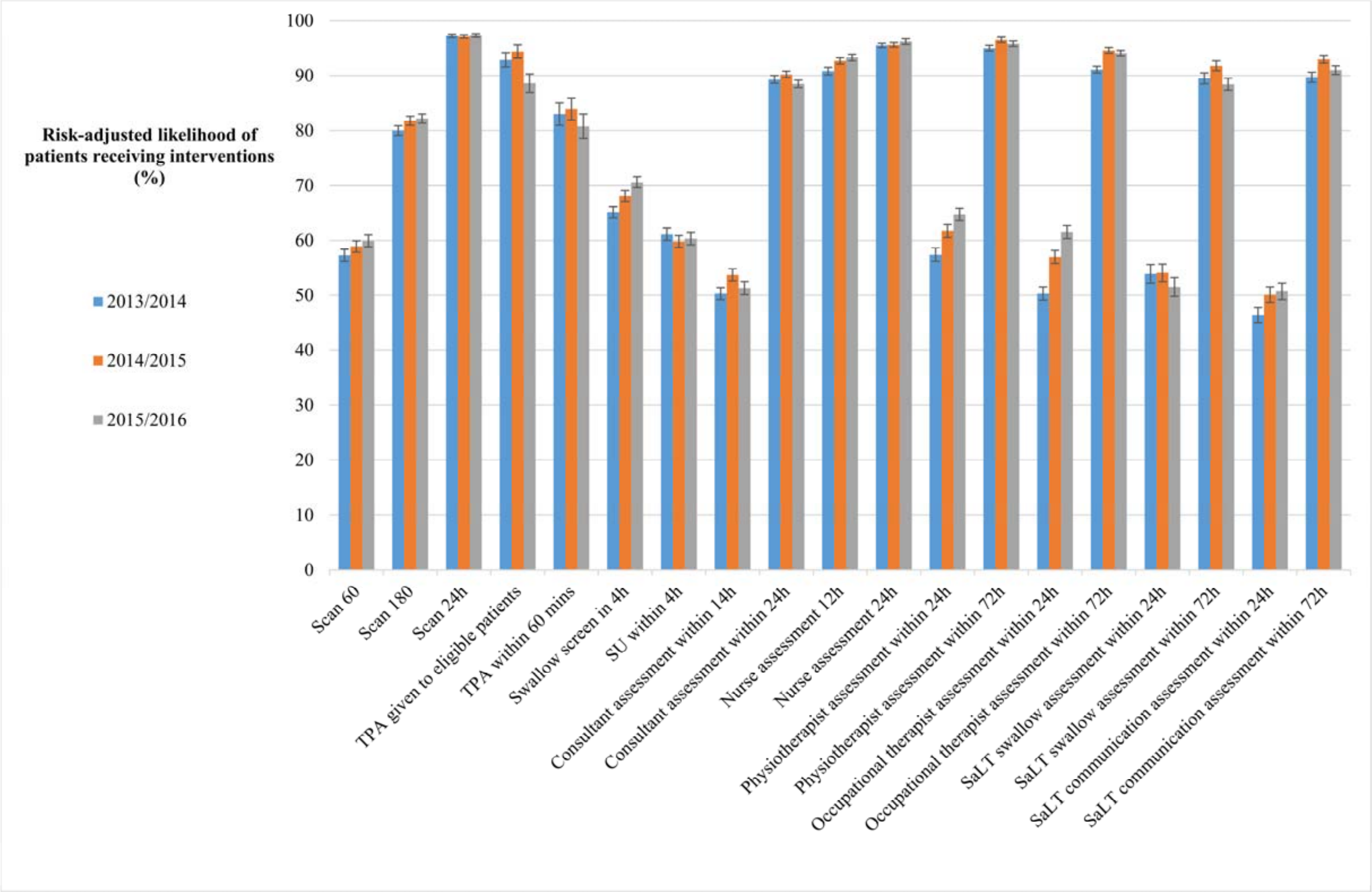

Figure C. Adjusted likelihood of patients receiving evidence-based clinical interventions in Rest of England

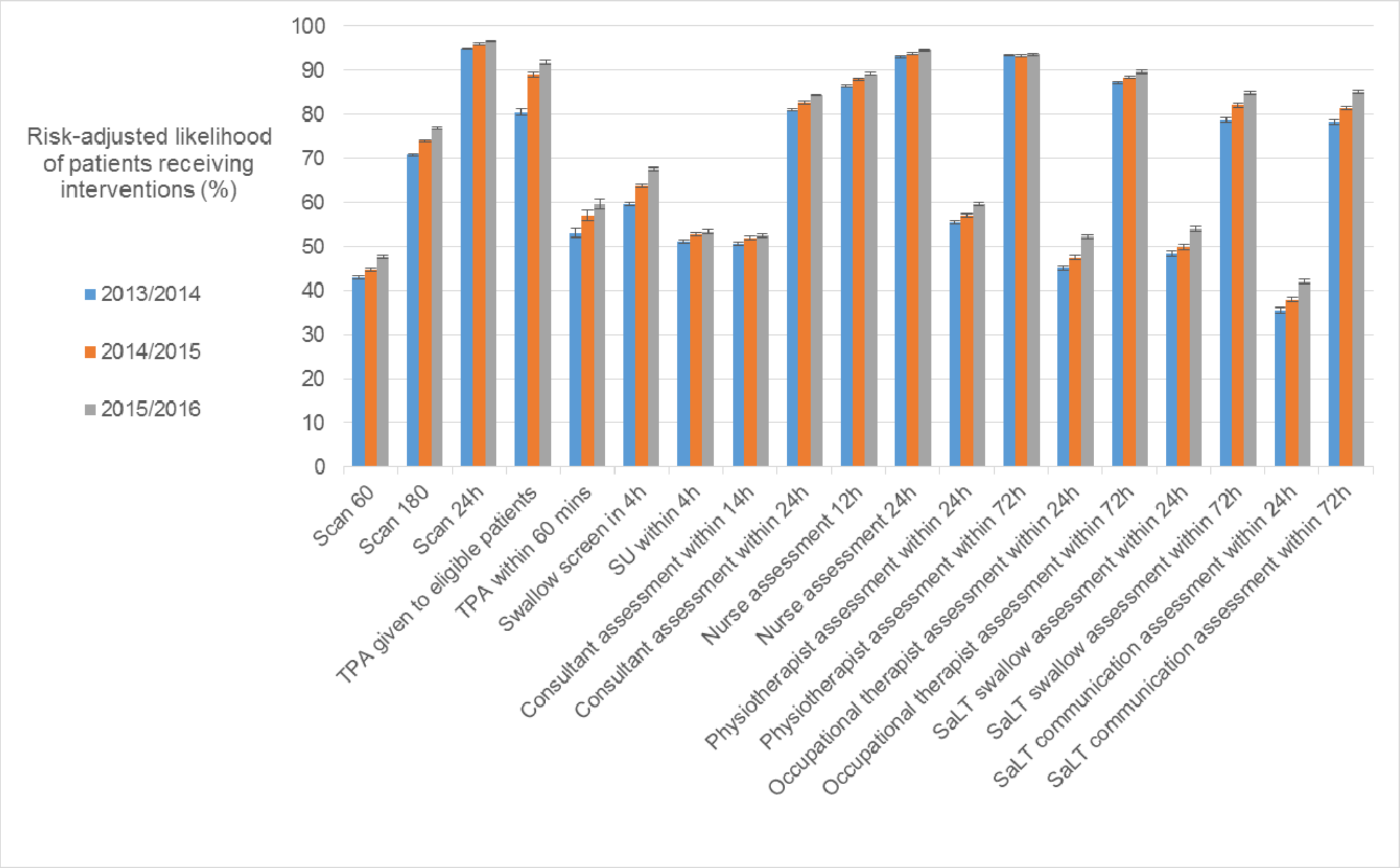

Supplement: Supplementary file 1 — Web appendix: Supplementary material [file mors046272.ww1.pdf]
